# Comparing Cutaneous Research Funded by the National Institute of Arthritis and Musculoskeletal and Skin Diseases with 2010 Global Burden of Disease Results

**DOI:** 10.1371/journal.pone.0102122

**Published:** 2014-07-08

**Authors:** Chante Karimkhani, Lindsay N. Boyers, David J. Margolis, Mohsen Naghavi, Roderick J. Hay, Hywel C. Williams, Luigi Naldi, Luc E. Coffeng, Martin A. Weinstock, Cory A. Dunnick, Hannah Pederson, Theo Vos, Christopher J. L. Murray, Robert P. Dellavalle

**Affiliations:** 1 College of Physicians and Surgeons, Columbia University, New York, New York, United States of America; 2 School of Medicine, Georgetown University, Washington, District of Columbia, United States of America; 3 Department of Biostatistics and Epidemiology and Dermatology, University of Pennsylvania, Philadelphia, Pennsylvania, United States of America; 4 Institute for Health Metrics and Evaluation, University of Washington, Seattle, Washington, United States of America; 5 Department of Dermatology, Kings College Hospital NHS Trust, London, United Kingdom; 6 Centre of Evidence Based Dermatology, University of Nottingham, Nottingham, United Kingdom; 7 Department of Dermatology, Azienda Ospedaliera papa Giovanni XXIII, Bergamo, Italy; 8 Dermatoepidemiology Unit, Veterans Affairs Medical Center Providence, Providence, Rhode Island, United States of America; 9 Department of Dermatology, Rode Island Hospital, Providence, Rhode Island, United States of America; 10 Departments of Dermatology and Epidemiology, Brown University, Providence, Rhode Island, United States of America; 11 Department of Dermatology, University of Colorado Anschutz Medical Campus, Aurora, Colorado, United States of America; 12 Dermatology Service, Unites States Department of Veterans Affairs, Eastern Colorado Health Care System, Denver, Colorado, United States of America; 13 University of Colorado School of Medicine, Aurora, Colorado, United States of America; 14 Department of Epidemiology, Colorado School of Public Health, University of Colorado Anschutz Medical Campus, Aurora, Colorado, United States of America; University of Tennessee, United States of America

## Abstract

**Importance:**

Disease burden data helps guide research prioritization.

**Objective:**

To determine the extent to which grants issued by the National Institute of Arthritis and Musculoskeletal and Skin Diseases (NIAMS) reflect disease burden, measured by disability-adjusted life years (DALYs) from Global Burden of Disease (GBD) 2010 project.

**Design:**

Two investigators independently assessed 15 skin conditions studied by GBD 2010 in the *NIAMS* database for grants issued in 2013. The 15 skin diseases were matched to their respective DALYs from GBD 2010.

**Setting:**

The United States NIAMS database and GBD 2010 skin condition disability data.

**Main Outcome(s) and Measure(s):**

Relationship of NIAMS grant database topic funding with percent total GBD 2010 DALY and DALY rank for 15 skin conditions.

**Results:**

During fiscal year 2013, 1,443 NIAMS grants were issued at a total value of $424 million. Of these grants, 17.7% covered skin topics. Of the total skin disease funding, 82% (91 grants) were categorized as “general cutaneous research.” Psoriasis, leprosy, and “other skin and subcutaneous diseases” (ie; immunobullous disorders, vitiligo, and hidradenitis suppurativa) were over-represented when funding was compared with disability. Conversely, cellulitis, decubitus ulcer, urticaria, acne vulgaris, viral skin diseases, fungal skin diseases, scabies, and melanoma were under-represented. Conditions for which disability and funding appeared well-matched were dermatitis, squamous and basal cell carcinoma, pruritus, bacterial skin diseases, and alopecia areata.

**Conclusions and Relevance:**

Degree of representation in *NIAMS* is partly correlated with DALY metrics. Grant funding was well-matched with disability metrics for five of the 15 studied skin diseases, while two skin diseases were over-represented and seven were under-represented. Global burden estimates provide increasingly transparent and important information for investigating and prioritizing national research funding allocations.

## Introduction

The 2010 Global Burden of Disease Study (GBD 2010) synthesizes data from 187 countries covering 291 diseases and injuries, 1160 sequelae, and 67 risk factors from 1990 to 2010 [1]. GBD 2010 measures disease burden in disability-adjusted life years (DALYs), a population health metric that combines mortality and morbidity by summing years of life lost and years lived with disability into one numerical value [2], [3]. Greater internal validity and compass distinguish GBD 2010 from previous work [2].

Research programs, policy makers, and healthcare providers all face the dilemma of fairly allocating limited resources [4], [5]. These stakeholders use data and criteria driven processes to determine priorities and reduce knowledge gaps [2], [6]. Epidemiological information and disease burden estimates contribute to these efforts.

The National Institute of Arthritis and Musculoskeletal and Skin Diseases (NIAMS), a division of the National Institutes of Health (NIH), supports research on the cause, treatment, and prevention of diseases of the bones, joints, muscles, and skin with US taxpayer dollars allocated from Congressional appropriations [7]–[9]. The skin focus of NIAMS research ranges from common diseases that affect millions of persons, such as eczema and psoriasis, to rare and overlooked diseases, such as pachyonychia congenita [7], [9]. Multifaceted and complex processes including expert and public comment guide NIAMS research priority setting, and a competitive peer-review system identifies the highest caliber research with the most potential [6]. This study compares current NIAMS funding of skin-focused research with skin disease burden estimated by GBD 2010.

## Methods

Fifteen skin conditions were studied by GBD 2010 under the umbrella category of *skin and subcutaneous diseases*: dermatitis (including eczema), acne vulgaris, bacterial skin diseases, viral skin diseases, urticaria, fungal skin diseases, pruritus, scabies, alopecia areata, cellulitis, decubitus ulcer, melanoma, psoriasis, squamous and basal cell carcinoma, and leprosy. In this study, squamous and basal cell carcinoma are collectively referred as non-melanoma skin cancer (NMSC). GBD 2010 also included an *other skin and subcutaneous diseases* category (see Table 1 for ICD-10 category definitions).

**Table 1:** Categorization of NIAMS grants, funding, and US Global Burden of Disease DALY metrics (arranged in order of decreasing US DALY).

Category	ICD-10 codes populating disease category in GBD 2010^a^	Funding^b^ (Percent)	Number of *NIAMS* grants in 2013	US DALY^c^ 2010 Absolute Number^d^ (Percent of total DALYs of all GBD conditions)	US DALY 2010 Skin Disease Rank^e^
Dermatitis including eczema	L20–L28	4,657,679.75 (6.35)	14	390,233 (0.48)	1
Non-melanoma skin cancer	C44, D04	5,750,690.5 (7.84)	24	230,918 (0.28)	2
Melanoma	C43, D03, D48.5	1,716,496.5 (2.34)	8	220,168 (0.27)	3
Acne vulgaris	L70	528,722.25 (0.72)	4	205,356 (0.25)	4
Pruritus	L29	2,435,743 (3.32)	10	134,569 (0.16)	5
Viral skin diseases	B00, B07–B09	0	0	116,972 (0.15)	6
Urticaria	L50	193,016 (0.26)	1	108,983 (0.14)	7
Decubitus ulcer	L89	156,387 (0.21)	1	84,763 (0.1)	8
Fungal skin diseases	B35, B36.0, B36.1, B36.2, B36.3, B36.8, B36.9	0	0	70,655 (0.086)	9
Psoriasis	L40–L41	4,558,347 (6.22)	19	64,342 (0.078)	10
Alopecia areata	L63.0, L63.1, L63.8, L63.9	362,137 (0.49)	1	58,662 (0.071)	11
Cellulitis	L03.0, L03.1, L03.2–L03.9	0	0	46,772 (0.057)	12
Bacterial skin diseases	L00, L01, L02, L04, L08, L88,L97, L98.0–L98.4	1,332,962 (1.82)	7	42,745 (0.054)	13
Scabies	B66	0	0	24,109 (0.029)	14
Leprosy	A30, B92	2,290,832 (3.12)	3	2.77 (0.0000034)	15
Other skin and subcutaneous diseases	B85, B87, B88, L05.0, L05.9, L10–L13, L28, L30, L42–L44, L51, L52–L53, L55–L60, L64–L68, L71–L75, L80–L85, L87, L90–L92, L93, L94–L95	9,612,761 (13.11)	44	240,645 (0.29)	N/A^f^
General cutaneous research	N/A	26,371,614 (35.96)	90	N/A	N/A
Conference	N/A	267,366 (0.36)	12	N/A	N/A
Training & department/institute program	N/A	7,391,817 (10.08)	22	N/A	N/A
Miscellaneous	N/A	5,708,836 (7.78)	10	N/A	N/A

a See reference 11.

b Only for fiscal year 2013; total funding for all NIAMS skin categories is $73,335,407.

c All ages.

d Rounded to the nearest integer.

e Out of the 15 skin disease categories studied by GBD 2010.

f N/A = not available.

All data were extracted independently by two authors (CK and LB) from January to February 2014 with consensus review by senior author (RPD) to resolve discrepancies. Grants awarded by NIAMS in 2013 were obtained online at http://report.nih.gov/award/index.cfm, by selecting “2013” for the fiscal year and “NIAMS” for the institute/center. Grant titles and abstracts were examined and categorized to determine if they focused on a skin condition. Skin-focused grants were selected and further classified (see categories listed in Table 1). The predominant focus and aim of the grant was used to determine its categorization. Isolated terms mentioned solely as *project terms*, *application*, or *public health relevance* were not used to guide categorization. Title and abstract terms leading to inclusion of the grant under one of the 15 skin conditions or the *other skin and subcutaneous diseases* category are defined in Tables S2 and S3 in File S1. Broad scientific themes of skin grant proposals, regardless of specific disease focus, were classified as *basic science* or *clinical research* (subcategories: *etiology*, *prevention*, *detection/diagnosis/treatment*) (see Table S1 in File S1). Grants were also placed into several additional categories not used by GBD 2010 including: training & department/institution program, conference, general cutaneous research, and miscellaneous (see Table S4 in File S1 for specific inclusion terms). The *general cutaneous research* category includes grants that lack a specific disease focus and the *miscellaneous* category includes dermatologic conditions not categorized by GBD 2010. If grants were assigned to more than one category, the grant amount was divided equally between the categories when summing funding totals. Grants with the same title but differing amounts of funding were counted separately but denoted by an asterisk (Table S1 in File S1).

Grants focusing on systemic conditions that also have skin manifestations were excluded, such as systemic sclerosis, systemic lupus erythematosus, and dermatomyositis. These three conditions are included under the GBD category of *musculoskeletal diseases*. However, variants of cutaneous lupus (discoid and subacute cutaneous lupus) were included in the *other skin and subcutaneous disease* category. Grants on wound healing were excluded since wound healing disability is not included as a skin condition by GBD.

The number of grants and proportion of NIAMS funding for each of the 15 skin diseases were matched to their respective disability, measured in disability-adjusted life years (DALYs). One DALY is equivalent to one lost year of healthy life [1]. Methods used by the GBD project to generate these disability estimates as well as GBD 2010 ICD-10 and ICD-9 code definitions for each disease have been previously described [10]–[12]. DALY metrics, expressed as percent of total US DALYs of all 291 conditions measured in GBD 2010, were obtained from the GBD Compare interactive time plot [13] Using this tool, we selected search parameters of ‘time plot,’ ‘DALYs’ metric, ‘United States’ place, ‘all ages,’ ‘both’ sexes, and ‘%’ units for each skin condition. Matching was accomplished by creating a data plot of funding versus disability to generate a linear line of best fit with correlation coefficient, and qualitatively determining those conditions that were well-matched or not well-matched.

This study did not involve human subjects, thus institutional review board approval was not necessary.

## Results

**Figure 1:**
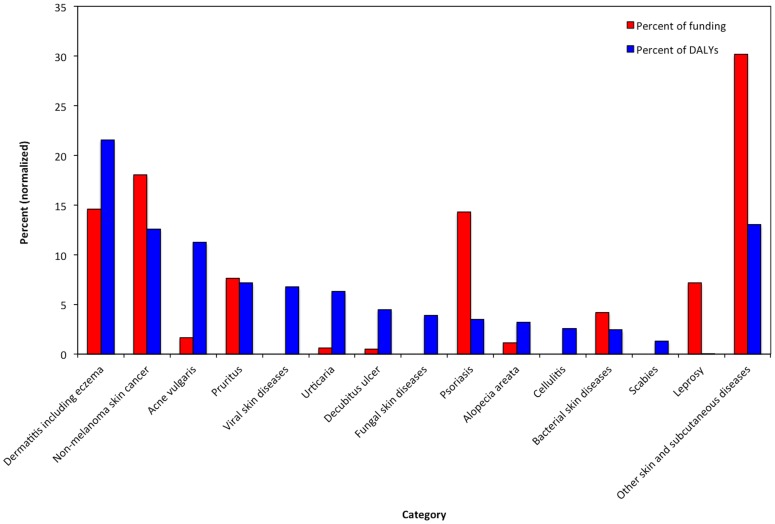
NIAMS skin funding in 2013 and skin disease disability bar graph—Distribution of NIAMS funding in 2013 for skin-related grants (red) compared to percent of total US GBD 2010 DALYs for each category (blue).

**Figure 2:**
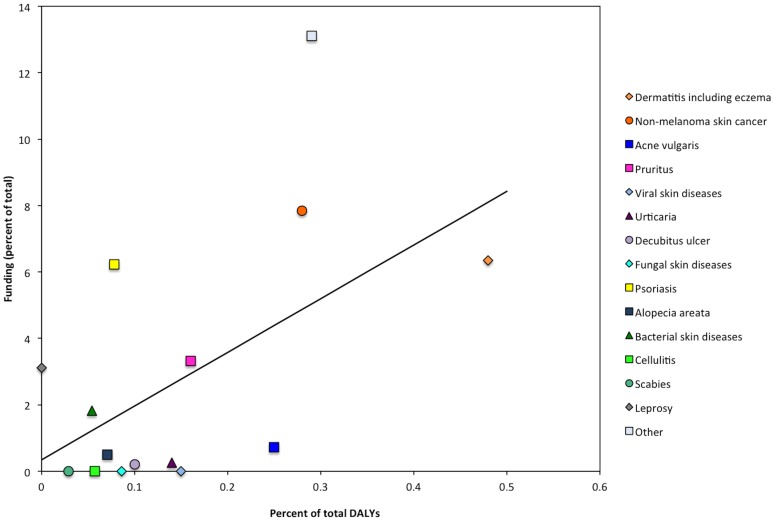
NIAMS skin funding in 2013 compared to skin disease disability scatter plot–GBD 2010 skin condition category NIAMS 2013 grant funding versus US GBD 2010 skin condition DALYs.

During fiscal year 2013, NIAMS issued 1,443 grants at a total value of $424 million, constituting 1.9% of the $22.5 billion issued for total grant funding by the NIH in 2013. Coincidentally, the overarching category of “skin and subcutaneous diseases” accounted for 1.9% of total US disability measured in GBD 2010. Amongst the 1,443 NIAMS grants, 256 grants (17.7%) pertained to skin topics, comprising $73.3 million (17.3% of total NIAMS funding in 2013) (Table S1 in File S1 for skin grant titles and categorization). The category of *general cutaneous research* comprising grants without a specific disease focus, received 36.0% of total skin funding and 90 grants. Comparing disability and funding, leprosy, psoriasis, and *other skin and subcutaneous diseases* demonstrated over-representation (Figures 1 and 2). Conversely, cellulitis, decubitus ulcer, urticaria, acne vulgaris, viral skin diseases, fungal skin diseases, scabies, and melanoma were under-represented. Conditions for which disability and funding appeared well-matched were dermatitis, NMSC, pruritus, bacterial skin diseases, and alopecia areata. Approximately 4.7% of skin-focused grants (n = 12) were assigned to more than one category.

Of the 15 specific GBD skin conditions, NMSC had the greatest representation (7.8% of total skin funding, 24 grants), which was well-matched with its second greatest US burden estimate (0.28% of total US DALY) (Table 1). Dermatitis had the greatest burden estimate of the 15 skin diseases (0.48% of total US DALY), ranking as the most disabling skin disease studied by GBD 2010. Dermatitis received the second greatest amount of funding of the 15 skin conditions (6.4% of total skin funding, 14 grants), followed by psoriasis (6.2%, 19 grants), pruritus (3.3%, 10 grants), and leprosy (3.1%, 3 grants).

Interestingly, acne vulgaris caused the 4^th^ greatest US skin disability (0.25% of total US DALYs) but received less funding (0.7% of total skin funding, 4 grants) than the 13^th^ most disabling category, bacterial skin diseases (1.8% of total skin funding, 7 grants. Similarly, melanoma was responsible for the 3^rd^ greatest US skin disability (0.27%) but received only 2.3% of total NIAMS skin funding (Table 1).

Of note, urticaria, decubitus ulcer, and alopecia areata were each represented by one grant and received 0.3%, 0.2%, and 0.5% of total skin funding, respectively. Disability metrics for these three conditions were 0.14%, 0.1%, and 0.071% of total US DALYs, respectively. Conversely, while leprosy had the lowest US DALY of the GBD 2010 skin conditions and accounted for a scant amount of the total US burden (0.0000034% of total US DALYs), the condition received 3.1% of total skin funding (3 grants). To put this in perspective, leprosy funding is similar to that of the 5^th^ most disabling skin disease, pruritus, which received 3.3% of total skin funding. GBD skin conditions with no grant funding or representation were viral skin diseases (0.15% of total US DALYs, DALY rank 9 of 15), fungal skin diseases (0.086%, rank 9), cellulitis (0.057%, rank 12), and scabies (0.029%, rank 14).

Eleven diseases within the umbrella *other skin and subcutaneous diseases* category had greater NIAMS representation (13.1% total skin funding, 44 grants) than any of the 15 individual GBD skin conditions (Table 2). This *other* category was more disabling than all studied skin conditions (0.29% of total US DALYs), with the exception of dermatitis. Within the *other skin and subcutaneous diseases* category, the immunobullous disorders (pemphigoid and pemphigus) received the greatest amount of total skin funding (4.9%), followed by vitiligo with 2.4% of total skin funding. Comparatively, the disabling but more common disease, hidradenitis suppurativa, received the lowest skin funding (0.1% of total skin funding, 1 grant).

**Table 2:** Conditions represented from the “other skin and subcutaneous diseases” Global Burden of Diseases category in the NIAMS 2013 skin-focused grants^a^ (arranged in order of decreasing funding).

Skin Condition	Funding^b^ (Percent)	Number of Grants
Immunobullous disorders	3,623,922.5 (4.94)	12
Vitiligo	1,739,205.5 (2.37)	8
Other epidermal thickening	1,616,370 (2.20)	5
Skin changes due to chronic exposure to nonionizing radiation	508,893.5 (0.69)	4
Hypertrophic disorders of skin	611,925 (0.83)	3
Lupus erythematosus	575,715 (0.79)	4
Primary cicatricial and scarring alopecia	458,442 (0.63)	2
Androgenic alopecia	59,339.25 (0.08)	1
Hypertrichosis	59,339.25 (0.08)	1
Other localized connective tissue disorders	276,959 (0.38)	3
Hidradenitis suppurativa	82,650 (0.11)	1

a See reference 11.

b Only for fiscal year 2013; total funding for all NIAMS skin categories is $73,345,407.

Twelve and 22 grants were devoted to conferences and training & department/institute programs, respectively (Table 1). Despite the high quantity of grant representation, only 0.4% of total skin funding was allocated to conferences while a larger proportion of 10.1% was allocated to training & department/institute programs. The miscellaneous category received 7.8% of total skin funding (10 grants) covering six skin conditions: pachyonychia congenita, port-wine stain, hemangioma, melanocytic nevi, and vesicant-induced skin injury.

Looking at broad scientific themes of grant proposal design, approximately 82 percent of skin-based NIAMS funding (209 grants) in 2013 was allocated to *basic science* grants. The remaining 47 non-basic science grants were clinical research grants investigating etiology (2 grants), prevention (3), and detection/diagnosis/treatment development (9), or devoted to training programs (13), establishment of research/CORE centers (8), or conferences (12) (Table 3).

**Table 3:** Broad scientific themes of NIAMS 2013 skin grants in percentage.

Category	Percent of total skin funding
Basic science	84.3
Clinical: etiology	0.6
Clinical: prevention	1.1
Clinical: detection/diagnosis/treatment	2.7
Training programs	3.3
Research/CORE center	7.5
Conferences	0.4

## Discussion

### Diseases for which NIAMS funding exceeded associated disability

Funding allocated to psoriasis, leprosy, and *other skin and subcutaneous diseases* over-matched the conditions' disability. Psoriasis is the most common autoimmune disease in the United States, affecting an estimated 7.5 million Americans [14]. Thus, while psoriasis' DALY was the sixth lowest amongst the 15 GBD skin conditions, it is not simply a skin problem. It has been shown to be an independent risk factor for cardiovascular disease and metabolic syndrome [15]. Psoriasis is responsible for an estimated 11.25 billion dollars in annual direct and indirect health care costs [16]. Many of the psoriasis NIAMS grants focused on study of the immune system for treatment options, correlating well with the evolution of novel treatment approaches over the past decade that target psoriasis' mechanistic origin in the immune system [17].

The three NIAMS grants included under leprosy are focused on the immunobiological aspects of leprosy. Although leprosy is scarce within the US, the condition remains endemic in regions of Angola, Brazil, the Central African Republic, India, Madagascar, Nepal and the United Republic of Tanzania and in previously highly endemic countries, such as the Democratic Republic of the Congo and Mozambique [18], [19]. Potential reasons for the apparent over-representation of NIAMS funds allocated to leprosy include contribution to global efforts for leprosy eradication, improvement of the US image abroad, applicability to other diseases, convenience of leprosy as a scientific model, and cultural implications of the disease [20]–[23].

While disability estimates are not available for the individual diseases in the *other skin and subcutaneous diseases* category, a curious hierarchy of funding exists. The greatest amount of funding in this category was devoted to the rare, autoimmune immunobullous disorders (pemphigoid and pemphigus). Vitiligo and other epidermal thickening followed with the second and third greatest funding dollars, respectively. Grant research proposals for these conditions focus heavily on pathogenesis and treatment. The least amount of funding in this category was allocated to hidradenitis suppurativa (HS). NIAMS funded one grant on treatment for HS, a common problem involving inflammation of the follicular epithelium that causes significant impact on quality of life [24].

### Diseases for which NIAMS funding proportion fell short of associated disability

In contrast to leprosy and psoriasis, acne vulgaris received disproportionately low funding compared to disability. Acne vulgaris is a very common skin diagnosis in the United States, affecting both teenagers and adults with a national price tag of 2.5 billion dollars [25]. A recent study revealed the need for large, randomized, controlled trials for acne treatment comparative effectiveness as well as the establishment of acne vulgaris standard treatment recommendations and training programs for medical students and residents [26]. Perhaps acne vulgaris is an area for future expansion in NIAMS-funded research.

Viral skin diseases, fungal skin diseases, scabies, and cellulitis were all uniformly under-represented by NIAMS. These infectious etiologies may be represented in the NIH infection branch, the National Institute of Allergy and Infectious Diseases (NIAID). However, the NIAMS specifically delineates that “studies of microbe-host interactions and of diseases triggered by bacterial, viral, or fungal infections, such as leprosy, acne, and post-herpetic neuralgia” are within their established funding research areas [27].

### Basic science focus

Over 80 percent of skin-based NIAMS funding (209 grants) in 2013 was allocated to *basic science* grants. The NIAMS does explicitly delineate basic science as “the foundation for tomorrow” as well as the importance of industry in “conducting basic research, developing new technologies, and commercializing federally supported discoveries” in their 2014 Statement to the Senate Appropriations Subcommittees [8]. While the NIAMS institutional focus has largely been on understanding the “molecular bases of about 4,000 diseases,” recent efforts have shifted to translate basic science discoveries to clinical applicability [28].

### Strengths and limitations

A 1999 cross-sectional study compared funding by the National Institutes of Health with burden of disease [29]. In response, NIH director Harold Varmus noted that “advocacy groups have tended, understandably, to focus their attention on alleged inequities between the toll of a specific disease and spending for research on that disease,” and further explained that “these claims are of great concern to the NIH, because they threaten to undermine the agency's credibility, and to Congress, because they challenge its oversight in a politically sensitive arena” [30]. The results of our study are not meant to criticize the funding levels of particular diseases. Instead the goal of our exploratory investigation is to examine how the most advanced method of measuring disease disability from GBD 2010 may potentially contribute to the multifaceted and complex funding prioritization. As Dr. Varmus further stated, “it is important to emphasize that there is not—and should not be—an absolute correspondence [between burden of disease and spending patterns]” [30].

The current study acknowledges the following limitations. NIAMS is one of 27 institutes and centers under the NIH, each devoted to specified areas of biomedical science. While percent funding shared between NIAMS and other NIH institutes would be informative, this data is not readily available. For instance, melanoma was included in the present study, however it should be noted that melanoma funding is shared between NIAMS and another NIH center, the National Cancer Institute (NCI) [31]. A future study investigating cancer funding, including melanoma, may be useful and informative.

The current study examined only grants issued by the NIAMS in fiscal year 2013. The 2013 Budget Control Act, also know as sequestration, had significant impact on the NIH, including the NIAMS, causing mandatory budgets cuts that resulted in a 5.6 percent decrease in the 2013 NIAMS budget ($505 million) [32]. Government shutdown in October 2013 led the NIH to temporarily furlough more than 75 percent of its employees and delay more than 200 grant review meetings across the NIH, resulting in decreased new and old grants awarded [32]. When money is tight, priorities naturally present themselves. Economic hardships such as the sequestration in 2013 allow a unique look into true research prioritization by a national research center.

The subjective nature of grant categorization must be mentioned. An objective and much easier method would involve inclusion of any grant that mentions a certain disease at least once in the title, abstract, or project terms under that particular disease category. However, it is common for a disease to be mentioned once as a project term or in the ‘public health relevance’ of the abstract but not part of the actual proposed research aims or methods. Thus, until more sophisticated search and categorization systems become available, categorization methods explained in the methods section are most appropriate. Finally, it should be noted that in general, NIH Institutes fund the “best science” and it is possible that under-represented areas were the result of proposals that did not meet funding criteria.

### Conclusions

This study mapped disability metrics from the GBD Study to research funded by the NIAMS. Burden of disease data appear to inform NIAMS prioritization, particularly for dermatitis, NMSC, pruritus, bacterial skin diseases, and alopecia areata. Multiple criteria including infrastructure and quality of research design, opportunity for scientific innovation, cost benefit, influence on vulnerable or neglected populations, interest group advocacy, disease transmissibility, public and patient impact, and predictions of future impact influence research funding. NIH funding is guided by the collaborative efforts of numerous agencies such as the Centers for Disease Control and Prevention, Department of Veterans Affairs, Department of Defense, and pharmaceutical industries. As the GBD database begins updating annually, ‘real-time’ information will be available for burden of disease in global and country-specific populations. While burden of disease should not serve as the sole factor determining funding allocation, funders should be aware of the burden of different skin diseases to inform and enhance a public discussion and optimize research prioritization.

## Supporting Information

File S1 **Supporting tables.** Table S1, Categorization of NIAMS 2013 skin-focused grants. Table S2, 15 Skin conditions studied by GBD 2010 and grant title or abstract terms supporting inclusion. Table S3, “Other skin and subcutaneous diseases” studied by GBD 2010 and grant title or abstract terms supporting inclusion. Table S4, Additional categories and grant title or abstract terms supporting inclusion.
(DOCX)
