# Supplementary material for: Comparing Cutaneous Research Funded by the National Institute of Arthritis and Musculoskeletal and Skin Diseases with 2010 Global Burden of Disease Results
Source: PLoS One. 2014 Jul 8;9(7):e102122. doi: 10.1371/journal.pone.0102122 (PMC4086973; doi:10.1371/journal.pone.0102122)
Supplement: File S1 — Supporting tables. Table S1, Categorization of NIAMS 2013 skin-focused grants. Table S2, 15 Skin conditions studied by GBD 2010 and grant title or abstract terms supporting inclusion. Table S3, “Other skin and subcutaneous diseases” studied by GBD 2010 and grant title or abstract terms supporting inclusion. Table S4, Additional categories and grant title or abstract terms supporting inclusion. (DOCX) [file pone.0102122.s001.docx]

**Table S1: Categorization of NIAMS 2013 skin-focused grants**

| **Institution** | **Grant Title** | **Category** | **Funding** | **Basic Science Grant?** |
| --- | --- | --- | --- | --- |
| Albany Medical College | [Keratinocyte Integrin Crosstalk During Wound Healing](http://projectreporter.nih.gov/project_info_description.cfm?icde=0&aid=8421453) | General cutaneous research | $327,125 | Yes |
| Baylor College of Medicine | [Regulation of skin epithelial stem cell homeostasis](http://projectreporter.nih.gov/project_info_description.cfm?icde=0&aid=8488414) | General cutaneous research | $314,982 | Yes |
| Benaroya Research Inst at Virginia Mason | [Regulation of TSLP-Mediated Skin Inflammation](http://projectreporter.nih.gov/project_info_description.cfm?icde=0&aid=8460069) | Dermatitis including eczema | $371,761 | Yes |
| Boston University Medical Campus | [Higher-order chromatin remodeling and regulation of pigmentation](http://projectreporter.nih.gov/project_info_description.cfm?icde=0&aid=8638589) | General cutaneous research | $208,718 | Yes |
|  | [Molecular control of normal and neoplastic skin stem cells](http://projectreporter.nih.gov/project_info_description.cfm?icde=0&aid=8487367) | General cutaneous research | $124,227 | Yes |
|  | [Bone Morphogentic Protein signaling in the control of skin development, hair grow](http://projectreporter.nih.gov/project_info_description.cfm?icde=0&aid=8492033) | General cutaneous research | $335,912 | Yes |
| Brigham and Women’s Hospital | [The clinical use of Flt3L - an immune adjuvant to potentiate Dendritic Cells](http://projectreporter.nih.gov/project_info_description.cfm?icde=0&aid=8494576) | General cutaneous research | $136,350 | Yes |
|  | [CD1 presentation of self glycolipids and lipopeptides to T cells](http://projectreporter.nih.gov/project_info_description.cfm?icde=0&aid=8460495) | General cutaneous research | $349,999 | Yes |
|  | [Mac-1 (CR3) and Fc gamma receptors in immune-mediated neutrophil cytotoxicity](http://projectreporter.nih.gov/project_info_description.cfm?icde=0&aid=8423361) | General cutaneous research | $359,430 | Yes |
|  | [Immune Evasion in Human Squamous Cell Carcinomas of the Skin](http://projectreporter.nih.gov/project_info_description.cfm?icde=0&aid=8502435) | Non-melanoma skin cancer  Other (Skin changes due to chronic exposure to nonionizing radiation) | $353,259 | No |
|  | [Dermatology Training Grant](http://projectreporter.nih.gov/project_info_description.cfm?icde=0&aid=8494571) | Training & department/institute program | $277,287 | No |
|  | [Skin Homing T Cells](http://projectreporter.nih.gov/project_info_description.cfm?icde=0&aid=8628602) | General cutaneous research | $438,194 | Yes |
| Brown University | [Ion Channel and Calcium Signaling in Ultraviolet Light Transduction in Human Skin](http://projectreporter.nih.gov/project_info_description.cfm?icde=0&aid=8612052) | General cutaneous research | $310,848 | Yes |
| Case Western Reserve University | [IL-17C mediated mechanisms of inflammation](http://projectreporter.nih.gov/project_info_description.cfm?icde=0&aid=8445590) | Psoriasis | $363,699 | Yes |
|  | [Neurogenic inflammation and psoriasiform dermatitis](http://projectreporter.nih.gov/project_info_description.cfm?icde=0&aid=8588226) | Psoriasis | $168,406 | Yes |
|  | [Psoriatic Regulatory T cell Dysfunction](http://projectreporter.nih.gov/project_info_description.cfm?icde=0&aid=8683592)* | Psoriasis | $53,620 | Yes |
|  | [PPAR-gamma Signaling in Normal Pilosebaceous Units and in Scarring Alopecia](http://projectreporter.nih.gov/project_info_description.cfm?icde=0&aid=8735236)* | Other (Primary cicatricial and scarring alopecia) | $139,500 | Yes |
|  | [NIAMS Core Center: Skin Diseases Research Center](http://projectreporter.nih.gov/project_info_description.cfm?icde=0&aid=8526374) | Training & department/institute program | $583,725 | No |
|  | [Psoriatic Regulatory T cell Dysfunction](http://projectreporter.nih.gov/project_info_description.cfm?icde=0&aid=8501377)* | Psoriasis | $322,164 | Yes |
|  | [PPAR-gamma Signaling in Normal Pilosebaceous Units and in Scarring Alopecia](http://projectreporter.nih.gov/project_info_description.cfm?icde=0&aid=8528334)* | Other (Primary cicatricial and scarring alopecia) | $318,942 | Yes |
|  | [Training in Investigative and Molecular Dermatology](http://projectreporter.nih.gov/project_info_description.cfm?icde=0&aid=8501373) | Training & department/institute program | $164,587 | No |
|  | [Amelioration of Vesicant-Induced Skin Injury by High Dose 25-Hydroxyvitamin D](http://projectreporter.nih.gov/project_info_description.cfm?icde=0&aid=8545673) | Miscellaneous | $788,987 | Yes |
| Children’s Hospital & Res Ctr at Oakland | [Cancer Associated Fibroblasts and Basal Cell Carcinoma Allografts](http://projectreporter.nih.gov/project_info_description.cfm?icde=0&aid=8468996) | Non-melanoma skin cancer | $76,950 | Yes |
| Children’s Hospital Corporation | [Regulators of Melanocyte and Melanoma Cell Identity](http://projectreporter.nih.gov/project_info_description.cfm?icde=0&aid=8460050) | Melanoma | $128,115 | Yes |
|  | [Hippo signaling and the control of epidermal development and growth](http://projectreporter.nih.gov/project_info_description.cfm?icde=0&aid=8545672) | Non-melanoma skin cancer | $371,925 | Yes |
| Columbia University Health Sciences | [Columbia University Medical Center Skin Disease Research Center (CUMCSDRC)](http://projectreporter.nih.gov/project_info_description.cfm?icde=0&aid=8499257) | Training & department/institute program | $596,614 | No |
|  | [Mechanisms of mechanosensory transduction in Merkel cells](http://projectreporter.nih.gov/project_info_description.cfm?icde=0&aid=8490308) | General cutaneous research | $327,068 | Yes |
| Cornell University | [Molecular Mechanisms of Cell Fate Decisions in Hair Follicle Stem Cells](http://projectreporter.nih.gov/project_info_description.cfm?icde=0&aid=8520181) | General cutaneous research | $347,428 | Yes |
| Duke University | [JUN PROTEINS IN EPIDERMAL HOMEOSTASIS AND NEOPLASIA](http://projectreporter.nih.gov/project_info_description.cfm?icde=0&aid=8471061) | Non-melanoma skin cancer | $322,164 | Yes |
|  | [Enhancing the CARRA: Integration and Dissemination of Clinical Data](http://projectreporter.nih.gov/project_info_description.cfm?icde=0&aid=8546978) | Training & department/institute program | $178,708 | No |
| Emory University | [Cadherin regulation in dermal endothelial cells](http://projectreporter.nih.gov/project_info_description.cfm?icde=0&aid=8526381) | General cutaneous research | $395,512 | Yes |
| Fred Hutchinson Can Res Ctr | [Mechanisms of epidermal growth during development, homeostasis, and tumorigenesis](http://projectreporter.nih.gov/project_info_description.cfm?icde=0&aid=8714189) | General cutaneous research | $249,000 | Yes |
| Glsynthesis, Inc. | [A Soft Topical Antiandrogenic Drug](http://projectreporter.nih.gov/project_info_description.cfm?icde=0&aid=8588704) | Dermatitis including eczema  Acne vulgaris  Other (Androgenic alopecia)  Other (Hypertrichosis) | $237,357 | Yes |
| Gordon Research Conferences | [2013 Collagen Gordon Research Conference and Gordon Research Seminar](http://projectreporter.nih.gov/project_info_description.cfm?icde=0&aid=8519773) | Conference | $20,000 | No |
|  | [2013 Epithelial Differentiation and Keratinization GRC/GRS](http://projectreporter.nih.gov/project_info_description.cfm?icde=0&aid=8522861) | Conference | $20,000 | No |
|  | [2013 Tissue Repair and Regeneration Gordon Research Conference](http://projectreporter.nih.gov/project_info_description.cfm?icde=0&aid=8528020) | Conference | $22,000 | No |
|  | [2013 Barrier Function of Mammalian Skin Gordon Research Conferences](http://projectreporter.nih.gov/project_info_description.cfm?icde=0&aid=8527924) | Conference | $15,000 | No |
| Hampton University | [Plan and Conduct the Hampton University Skin of Color Research Institute Skin of](http://projectreporter.nih.gov/project_info_description.cfm?icde=0&aid=8597667) | Conference | $15,000 | No |
| Harvard University (Medical School) | [Mechanisms of sensory neuron control over skin immune responses](http://projectreporter.nih.gov/project_info_description.cfm?icde=0&aid=8521056) | Psoriasis | $33,923 | Yes |
|  | [Molecular and Cellular Mechanisms of Vascular Anomalies](http://projectreporter.nih.gov/project_info_description.cfm?icde=0&aid=8528325) | Miscellaneous | $1,457,109 | Yes |
| Henry M. Jackson FDN for Adv Mil/Med | [Regulation of Dermal Fibroblasts in Skin Regeneration and Healing](http://projectreporter.nih.gov/project_info_description.cfm?icde=0&aid=8529460) | General cutaneous research | $320,808 | Yes |
| Indiana Univ-Purdue Univ at Indianapolis | [The Indiana Cutaneous Biological Research Training Program](http://projectreporter.nih.gov/project_info_description.cfm?icde=0&aid=8473519) | Training & department/institute program | $131,407 | No |
| Jackson Laboratory | [Discovering Novel Gene Networks for Skin Diseases](http://projectreporter.nih.gov/project_info_description.cfm?icde=0&aid=8582268) | General cutaneous research | $191,997 | Yes |
|  | [Genetics of Alopecia Areata in the C3H/HeJ Mouse](http://projectreporter.nih.gov/project_info_description.cfm?icde=0&aid=8506975) | Alopecia areata | $362,137 | Yes |
| John Wayne Cancer Institute | [Mechanisms of Innate and Acquired Host Defense and Tissue Injury in Skin](http://projectreporter.nih.gov/project_info_description.cfm?icde=0&aid=8500211) | Leprosy | $378,184 | Yes |
| Johns Hopkins University | [Cytokine Regulation of Wound Induced Skin Regeneration](http://projectreporter.nih.gov/project_info_description.cfm?icde=0&aid=8457180) | General cutaneous research | $57,734 | Yes |
|  | [Interferons and cytotoxic lymphocytes in dermatomyositis and cutaneous lupus](http://projectreporter.nih.gov/project_info_description.cfm?icde=0&aid=8725793) | Other (Lupus erythematosus) | $200,001 | Yes |
|  | [The structural support role of keratins in skin](http://projectreporter.nih.gov/project_info_description.cfm?icde=0&aid=8386927) | General cutaneous research | $456,127 | Yes |
|  | [Keratins, Wound Reepithelialization, and Hair Follicle Cycling](http://projectreporter.nih.gov/project_info_description.cfm?icde=0&aid=8452599) | General cutaneous research | $426,636 | Yes |
|  | [Transgenic Regulation of Keratinocyte to Nociceptor Signaling](http://projectreporter.nih.gov/project_info_description.cfm?icde=0&aid=8449204) | General cutaneous research | $173,138 | Yes |
| Kaiser Foundation Research Institute | [Comparative Effectiveness of Field-Treatments for Multiple Actinic](http://projectreporter.nih.gov/project_info_description.cfm?icde=0&aid=8447336) keratoses | Non-melanoma skin cancer  Other (Skin changes due to chronic exposure to nonionizing radiation) | $78,966 | No |
| Loyola University Chicago | [Modulating tolerance in a spontaneous mouse model of autoimmune vitiligo](http://projectreporter.nih.gov/project_info_description.cfm?icde=0&aid=8457139) | Other (Vitiligo) | $305,132 | Yes |
|  | [Linking Nicotinic Activation with Skin Innate Immunity and Atopic Dermatitis](http://projectreporter.nih.gov/project_info_description.cfm?icde=0&aid=8460051) | Dermatitis including eczema | $319,556 | Yes |
| Massachusetts General Hosp | [Epigenetic regulation of epidermal homeostasis](http://projectreporter.nih.gov/project_info_description.cfm?icde=0&aid=8584899) | General cutaneous research | $337,466 | Yes |
|  | [7th World Congress on Itch (WCI)](http://projectreporter.nih.gov/project_info_description.cfm?icde=0&aid=8597572) | Pruritus  Conference | $15,000 | No |
|  | [The MITF transcriptional network: melanoma risk and carcinogenesis](http://projectreporter.nih.gov/project_info_description.cfm?icde=0&aid=8439860) | Melanoma | $342,200 | Yes |
|  | [Growth/differentiation control of keratinocytes by ROR alpha](http://projectreporter.nih.gov/project_info_description.cfm?icde=0&aid=8451403) | General cutaneous research | $110,376 | Yes |
|  | [Growth differentiation control in primary keratinocytes](http://projectreporter.nih.gov/project_info_description.cfm?icde=0&aid=8459430) | General cutaneous research | $431,247 | Yes |
|  | [Inductive properties of the dermal papilla of the hair follicle](http://projectreporter.nih.gov/project_info_description.cfm?icde=0&aid=8519305) | General cutaneous research | $360,840 | Yes |
|  | [Itch, proteases and protease-activated receptors](http://projectreporter.nih.gov/project_info_description.cfm?icde=0&aid=8501380) | Pruritus | $360,436 | Yes |
|  | [The Role of Presenilin in Hidradenitis Suppurativa](http://projectreporter.nih.gov/project_info_description.cfm?icde=0&aid=8511572) | Other (Hidradenitis suppurativa) | $82,650 | Yes |
| Mayo Clinic Arizona | [Eosinophil-nerve Interactions in Mouse Models of Dermatitis](http://projectreporter.nih.gov/project_info_description.cfm?icde=0&aid=8448622) | Dermatitis including eczema | $558,283 | Yes |
| Medical College of Wisconsin | [Function and Targeting of CCR6/CCL20 in Autoimmune Psoriasiform Skin Disease](http://projectreporter.nih.gov/project_info_description.cfm?icde=0&aid=8502109) | Psoriasis | $314,313 | Yes |
|  | [Pediatric Dermatology Research Alliance Annual Conference](http://projectreporter.nih.gov/project_info_description.cfm?icde=0&aid=8597492) | Conference | $59,616 | No |
| Montana State University – Bozeman | [staphylococcus aureus biofilms mediate keratinocyte apoptosis](http://projectreporter.nih.gov/project_info_description.cfm?icde=0&aid=8496719) | Bacterial skin diseases | $68,400 | Yes |
| Mount Sinai School of Medicine | [Exploring How Dermal Papilla Precursors Regulate Hair Follicle Formation](http://projectreporter.nih.gov/project_info_description.cfm?icde=0&aid=8579382) | General cutaneous research | $359,102 | Yes |
|  | [A Study of ILV-94 (Anti-22 Antibody) Administered via IV in Atopic Dermatitis](http://projectreporter.nih.gov/project_info_description.cfm?icde=0&aid=8580222) | Dermatitis including eczema | $631,536 | Yes |
|  | [Elucidating the Functions of Epigenetic Regulators in Control of Skin Stem Cells](http://projectreporter.nih.gov/project_info_description.cfm?icde=0&aid=8434079) | General cutaneous research | $236,550 | Yes |
|  | [Specification of Dermal Papilla Cell Fate in the Hair Follicle Stem Cell Niche](http://projectreporter.nih.gov/project_info_description.cfm?icde=0&aid=8437212) | General cutaneous research | $345,010 | Yes |
|  | [Role of Chromatin Regulators in Skin Control](http://projectreporter.nih.gov/project_info_description.cfm?icde=0&aid=8541696) | General cutaneous research | $362,306 | Yes |
| National Jewish Health | [The Role of Bacterial Toxins in Human Skin Disease](http://projectreporter.nih.gov/project_info_description.cfm?icde=0&aid=8508065) | Dermatitis including eczema  Bacterial skin diseases | $292,718 | Yes |
| Nevus Outreach, Inc. | [2013 International Expert Meeting on Congenital Melanocytic Nevi (CMN) and Neuroc](http://projectreporter.nih.gov/project_info_description.cfm?icde=0&aid=8597737) | Conference  Miscellaneous | $30,000 | No |
| New York University School of Medicine | [Mechanisms of Homeostasis and Invasive Cell Migration in Skin Tumorigenesis](http://projectreporter.nih.gov/project_info_description.cfm?icde=0&aid=8731049)* | Non-melanoma skin cancer | $101,225 | Yes |
|  | [Biogenesis of Melanosomes](http://projectreporter.nih.gov/project_info_description.cfm?icde=0&aid=8698892)* | Melanoma  Other (Vitiligo) | $172,288 | Yes |
|  | [The regulation of melanocyte stem cells by Wnt signaling](http://projectreporter.nih.gov/project_info_description.cfm?icde=0&aid=8699417) | General cutaneous research | $160,561 | Yes |
|  | [Mechanisms of Homeostasis and Invasive Cell Migration in Skin Tumorigenesis](http://projectreporter.nih.gov/project_info_description.cfm?icde=0&aid=8517009)* | Non-melanoma skin cancer | $236,549 | Yes |
|  | [Biogenesis of Melanosomes](http://projectreporter.nih.gov/project_info_description.cfm?icde=0&aid=8438393)* | Melanoma  Other (Vitiligo) | $430,695 | Yes |
|  | [The Pharmacology of Dermal Fibrosis](http://projectreporter.nih.gov/project_info_description.cfm?icde=0&aid=8401849) | Other (Hypertrophic disorders of skin) | $336,685 | Yes |
|  | [The regulation of melanocyte stem cells by Wnt signaling](http://projectreporter.nih.gov/project_info_description.cfm?icde=0&aid=8509604)* | General cutaneous research | $361,238 | Yes |
| Northern California Institute/Res/Edu | [Role of vitamin D receptor in DNA repair](http://projectreporter.nih.gov/project_info_description.cfm?icde=0&aid=8569938) | General cutaneous research | $188,955 | Yes |
|  | [Vitamin D Receptor Coactivators in Keratinocytes](http://projectreporter.nih.gov/project_info_description.cfm?icde=0&aid=8488410) | General cutaneous research | $304,106 | Yes |
|  | [Calcium-sensing Receptor and Keratinocyte Differentiation](http://projectreporter.nih.gov/project_info_description.cfm?icde=0&aid=8436129) | General cutaneous research | $304,106 | Yes |
|  | [Melanocyte-Keratinocyte Cross-Talk In Relation To Barrier Function](http://projectreporter.nih.gov/project_info_description.cfm?icde=0&aid=8471653) | General cutaneous research | $304,106 | Yes |
|  | [Pathogenesis and Therapy of Ichthyosis in Disorders of Lipid Metabolism](http://projectreporter.nih.gov/project_info_description.cfm?icde=0&aid=8434177) | Other (Other epidermal thickening) | $316,778 | Yes |
|  | [S1P-Mediated Stimulation of Antimicrobial Defense](http://projectreporter.nih.gov/project_info_description.cfm?icde=0&aid=8507601) | Dermatitis including eczema  Bacterial skin diseases | $316,778 | Yes |
|  | [The Lipid and Tight Junction Epidermal Barriers are Interdependent](http://projectreporter.nih.gov/project_info_description.cfm?icde=0&aid=8511569) | Bacterial skin diseases | $158,389 | Yes |
| Northwestern University at Chicago | [Northwestern University Skin Disease Research Core Center](http://projectreporter.nih.gov/project_info_description.cfm?icde=0&aid=8492036) | Training & department/institute program | $579,500 | No |
|  | [Function of Desmoglein 1/Pemphigus Foliaceus Antigen](http://projectreporter.nih.gov/project_info_description.cfm?icde=0&aid=8515331) | Other (Other epidermal thickening) | $447,154 | Yes |
|  | [Functional and Structural Links between Cadherin, Gamma-Secretase, and Notch](http://projectreporter.nih.gov/project_info_description.cfm?icde=0&aid=8469391) | General cutaneous research | $312,930 | Yes |
|  | [Topical Delivery of siRNA Nanconjugates: Suppressing Epidermal Hyperplasia](http://projectreporter.nih.gov/project_info_description.cfm?icde=0&aid=8433345) | General cutaneous research | $325,066 | Yes |
|  | [Epha/Ephrin - A Signaling in Epidermal Differentiation and Disease](http://projectreporter.nih.gov/project_info_description.cfm?icde=0&aid=8541694) | General cutaneous research | $324,896 | Yes |
|  | [Desmoplakin Assembly and Function in Epidermis](http://projectreporter.nih.gov/project_info_description.cfm?icde=0&aid=8451594) | General cutaneous research | $441,823 | Yes |
|  | [Post Graduate Program in Cutaneous Biology](http://projectreporter.nih.gov/project_info_description.cfm?icde=0&aid=8448620) | Training & department/institute program | $109,847 | No |
| Oregon Health & Science University | [The Role of the Skin Barrier in Atopic Dermatitis Development and Prevention](http://projectreporter.nih.gov/project_info_description.cfm?icde=0&aid=8604539)* | Dermatitis including eczema | $994 | No |
|  | [The Role of the Skin Barrier in Atopic Dermatitis Development and Prevention](http://projectreporter.nih.gov/project_info_description.cfm?icde=0&aid=8451395)* | Dermatitis including eczema | $127,980 | No |
|  | [Trim32 Regulation of Piasy in Skin Homeostasis](http://projectreporter.nih.gov/project_info_description.cfm?icde=0&aid=8461180) | Psoriasis | $312,848 | Yes |
|  | [Montagna Symposium on the Biology of Skin](http://projectreporter.nih.gov/project_info_description.cfm?icde=0&aid=8471056) | Conference | $38,000 | No |
| Pachyonychia Congenita Project | [2013 International Pachyonychia Congenita Consortium (IPCC) Symposium](http://projectreporter.nih.gov/project_info_description.cfm?icde=0&aid=8636235) | Conference  Miscellaneous | $12,500 | No |
| Pennsylvania State University | [Development of Clinical Trials Outcome Instruments for Acne Vulgaris](http://projectreporter.nih.gov/project_info_description.cfm?icde=0&aid=8580866) | Acne vulgaris | $204,113 | No |
| Princeton University | [Regulation of Cell Polarity During Epidermal Growth and Homeostasis](http://projectreporter.nih.gov/project_info_description.cfm?icde=0&aid=8529194) | General cutaneous research | $236,550 | Yes |
| RHBS-Robert Wood Johnson Medical School | [UMDNJ/Rutgers University CounterACT Research Center of Excellence](http://projectreporter.nih.gov/project_info_description.cfm?icde=0&aid=8545525) | Training & department/institute program  Miscellaneous | $2,369,028 | No |
| Rockefeller University | [Regulation of Quiescence and Activation in Skin Stem Cells](http://projectreporter.nih.gov/project_info_description.cfm?icde=0&aid=8509979) | General cutaneous research | $96,471 | Yes |
|  | [Mechanisms used by skin dendritic cells to induce regulatory T cells](http://projectreporter.nih.gov/project_info_description.cfm?icde=0&aid=8460099) | General cutaneous research | $95,593 | Yes |
|  | [Role of Primary Cilia During Epidermal Morphogenesis](http://projectreporter.nih.gov/project_info_description.cfm?icde=0&aid=8494575) | General cutaneous research | $95,796 | Yes |
|  | [Regulation of Epidermal Development and Differentiation](http://projectreporter.nih.gov/project_info_description.cfm?icde=0&aid=8471057) | General cutaneous research | $573,687 | Yes |
|  | [Skin Stem Cells: Purification and Characterization](http://projectreporter.nih.gov/project_info_description.cfm?icde=0&aid=8461613) | General cutaneous research | $343,320 | Yes |
|  | [Origin and Function of Inflammatory Dendritic Cells in Psoriasis.](http://projectreporter.nih.gov/project_info_description.cfm?icde=0&aid=8510576) | Psoriasis | $362,306 | Yes |
|  | [Cell adhesion and cytoskeletal dynamics in skin](http://projectreporter.nih.gov/project_info_description.cfm?icde=0&aid=8386937) | General cutaneous research | $730,724 | Yes |
| Scarless Laboratories, Inc. | [A novel anti-scar peptide for cutaneous wound repair](http://projectreporter.nih.gov/project_info_description.cfm?icde=0&aid=8455289) | Other (Hypertrophic disorders of skin) | $199,740 | Yes |
| Scripps Research Institute | [ATP in Skin Immunity](http://projectreporter.nih.gov/project_info_description.cfm?icde=0&aid=8581622) | Other (Skin changes due to chronic exposure to nonionizing radiation) | $122,850 | Yes |
| Signum Biosciences | [A Topical Non-steroidal Anti-inflammatory for Atopic Dermatitis](http://projectreporter.nih.gov/project_info_description.cfm?icde=0&aid=8450681) | Dermatitis including eczema | $998,971 | No |
| Sloan-Kettering Inst Can Res | [The role of melanocyte precursors in zebrafish pigmentation disorders](http://projectreporter.nih.gov/project_info_description.cfm?icde=0&aid=8423391) | Melanoma | $126,900 | Yes |
|  | [THE FRAMINGHAM SCHOOL STUDY OF NEVI IN CHILDREN SONIC II](http://projectreporter.nih.gov/project_info_description.cfm?icde=0&aid=8468117) | Miscellaneous | $669,406 | No |
| Society for Investigative Dermatology | [SID Retreat for Future Academicians](http://projectreporter.nih.gov/project_info_description.cfm?icde=0&aid=8462203) | Conference | $25,000 | No |
| Stanford University | [Characterization of Homeostatic Regulators Disrupted in Epidermal Neoplasia](http://projectreporter.nih.gov/project_info_description.cfm?icde=0&aid=8562474) | Non-melanoma skin cancer | $125,604 | Yes |
|  | [Homeostatic Regulators Disrupted in Skin Carcinogenesis](http://projectreporter.nih.gov/project_info_description.cfm?icde=0&aid=8503150) | Non-melanoma skin cancer | $330,543 | Yes |
|  | [Regulating Gli Function in Hair Follicle Progenitors](http://projectreporter.nih.gov/project_info_description.cfm?icde=0&aid=8647650) | Non-melanoma skin cancer | $340,401 | Yes |
|  | [Regulation of human skin homeostasis by histone arginine methylation regulators](http://projectreporter.nih.gov/project_info_description.cfm?icde=0&aid=8448767) | General cutaneous research | $55,670 | Yes |
|  | [Gene Regulatory Mechanisms of Epidermal Growth](http://projectreporter.nih.gov/project_info_description.cfm?icde=0&aid=8464523) | General cutaneous research | $403,274 | Yes |
|  | [Stromal Regulation of Basal Cell Carcinoma Formation](http://projectreporter.nih.gov/project_info_description.cfm?icde=0&aid=8460142) | Non-melanoma skin cancer | $344,324 | Yes |
|  | [Laminins in the Skin](http://projectreporter.nih.gov/project_info_description.cfm?icde=0&aid=8467994) | General cutaneous research | $326,571 | Yes |
|  | [Signaling Regulators of Epithelial Homeostasis and Neoplasia](http://projectreporter.nih.gov/project_info_description.cfm?icde=0&aid=8464527) | Non-melanoma skin cancer | $324,157 | Yes |
|  | [Gene Transfer for Recessive Dystrophic Epidermolysis Bullosa](http://projectreporter.nih.gov/project_info_description.cfm?icde=0&aid=8529189) | Other (Pemphigoid) | $615,434 | Yes |
|  | [Postgraduate Training Program in Epithelial Biology](http://projectreporter.nih.gov/project_info_description.cfm?icde=0&aid=8468992) | Training & department/institute program | $157,528 | No |
| State University New York Stony Brook | [Cx26 mutations in syndromic deafness linked to skin disease](http://projectreporter.nih.gov/project_info_description.cfm?icde=0&aid=8438425) | General cutaneous research | $320,405 | Yes |
|  | [Planar Cell Polarity Signaling in Hair Follicle Formation](http://projectreporter.nih.gov/project_info_description.cfm?icde=0&aid=8515204) | General cutaneous research | $334,368 | Yes |
| State University of New York at Buffalo | [Novel Genetic Models to Study the Role of DNp63 in Squamous Cell Carcinoma](http://projectreporter.nih.gov/project_info_description.cfm?icde=0&aid=8585388) | Non-melanoma skin cancer | $79,500 | Yes |
| Sunny Biodiscovery, Inc. | [Novel Skin Protectant Retinoid for the Treatment of Psoriasis](http://projectreporter.nih.gov/project_info_description.cfm?icde=0&aid=8449026) | Psoriasis | $149,451 | Yes |
| SUNY Downstate Medical Center | [Mouse nude locus and skin development](http://projectreporter.nih.gov/project_info_description.cfm?icde=0&aid=8490304) | General cutaneous research | $324,021 | Yes |
| Surface Bioadvances, Inc. | [Bacterial fermentation in skin microbiome as probiotics (Bfismp) against S. aureu](http://projectreporter.nih.gov/project_info_description.cfm?icde=0&aid=8452574) | Bacterial skin diseases | $150,000 | Yes |
| Thomas Jefferson University | [Mechanism of skin-specific targeting of adult stem cells](http://projectreporter.nih.gov/project_info_description.cfm?icde=0&aid=8630229) | Other (Pemphigoid) | $329,375 | Yes |
|  | [Role of Desmosomal Adhesion in Carcinogenesis](http://projectreporter.nih.gov/project_info_description.cfm?icde=0&aid=8386930) | Non-melanoma skin cancer | $317,034 | Yes |
|  | [Mineralization/Anti-Mineralization Networks in the Skin](http://projectreporter.nih.gov/project_info_description.cfm?icde=0&aid=8509607) | Other (Other localized connective tissue disorders) | $150,587 | Yes |
|  | [TRAINING IN MOLECULAR DERMATOLOGY AND CUTANEOUS CONNECTIVE TISSUE DISEASES](http://projectreporter.nih.gov/project_info_description.cfm?icde=0&aid=8461621) | Training & department/institute program | $76,161 | No |
| Transderm, Inc. | [Pachyonychia congenita clinical trial using therapeutic self-delivery siRNAs](http://projectreporter.nih.gov/project_info_description.cfm?icde=0&aid=8530953) | Miscellaneous | $1,045,754 | Yes |
| Univ of Massachusetts Med Sch Worchester | [Use of comparative genomics to identify novel regulators of melanoma progression](http://projectreporter.nih.gov/project_info_description.cfm?icde=0&aid=8579385) | Melanoma | $353,813 | Yes |
|  | [Targeting IFN gamma and chemokines to treat vitiligo in a humanized mouse model](http://projectreporter.nih.gov/project_info_description.cfm?icde=0&aid=8502440) | Other (Vitiligo) | $123,660 | Yes |
| Univ of North Carolina Chapel Hill | [Etiology and Pathogenesis of Pemphigus](http://projectreporter.nih.gov/project_info_description.cfm?icde=0&aid=8579261) | Other (Pemphigus) | $323,000 | Yes |
|  | [Interactions of lgC4 & lgE anti-Dsg1 Autoantibodies in Endemic Pemphigus Foliaceu](http://projectreporter.nih.gov/project_info_description.cfm?icde=0&aid=8693332)* | Other (Pemphigus) | $39,793 | Yes |
|  | [Interactions of lgC4 & lgE anti-Dsg1 Autoantibodies in Endemic Pemphigus Foliaceu](http://projectreporter.nih.gov/project_info_description.cfm?icde=0&aid=8522259)* | Other (Pemphigus) | $109,322 | Yes |
|  | [Role of the matrix metalloproteinase in pemphigus autoantibody-mediated epidermal](http://projectreporter.nih.gov/project_info_description.cfm?icde=0&aid=8478045) | Other (Pemphigus) | $284,715 | Yes |
| University of Alabama at Birmingham | [UAB Skin Diseases Research Center](http://projectreporter.nih.gov/project_info_description.cfm?icde=0&aid=8538747) | Training & department/institute program | $485,498 | No |
|  | [Therapeutic Intervention of Lewisite-Mediated Cutaneous Blistering-Inflammation](http://projectreporter.nih.gov/project_info_description.cfm?icde=0&aid=8544981) | Miscellaneous | $366,251 | Yes |
| University of Arizona | [GILT and regulation of Treg development in cutaneous autoimmunity](http://projectreporter.nih.gov/project_info_description.cfm?icde=0&aid=8582162) | Other (Vitiligo) | $75,750 | Yes |
| University of California | [Epigenetic Regulators in Epidermal Homeostasis and Neoplasia](http://projectreporter.nih.gov/project_info_description.cfm?icde=0&aid=8386924) | Non-melanoma skin cancer | $125,361 | Yes |
|  | [Genetic Influence in Pediatric Psoriasis](http://projectreporter.nih.gov/project_info_description.cfm?icde=0&aid=8509605) | Psoriasis | $132,300 | Yes |
|  | [Proteolysis and Skin Antimicrobials](http://projectreporter.nih.gov/project_info_description.cfm?icde=0&aid=8434753) | Bacterial skin diseases | $331,313 | Yes |
|  | [UCSD Dermatologist Investigator Training Program](http://projectreporter.nih.gov/project_info_description.cfm?icde=0&aid=8451411) | Training & department/institute program | $197,548 | No |
| University of California Berkeley | [Roles and functions of ion channels that mediate mammalian touch transduction.](http://projectreporter.nih.gov/project_info_description.cfm?icde=0&aid=8461909) | General cutaneous research | $320,817 | Yes |
| University of California Davis | [Warmth enhancement of itch via TRPV4](http://projectreporter.nih.gov/project_info_description.cfm?icde=0&aid=8509420) | Pruritus | $87,885 | Yes |
|  | [Galectin-3 in regulation of allergic skin inflammation](http://projectreporter.nih.gov/project_info_description.cfm?icde=0&aid=8401847) | Dermatitis including eczema | $314,384 | Yes |
|  | [Neural mechanisms of itch](http://projectreporter.nih.gov/project_info_description.cfm?icde=0&aid=8500209) | Pruritus | $312,848 | Yes |
| University of California Irvine | [Molecular mechanisms of pulsed dye laser combined with topical rapamycin for port](http://projectreporter.nih.gov/project_info_description.cfm?icde=0&aid=8425982) | Miscellaneous | $88,700 | No |
|  | [Phosphoinositide Signaling Regulates Melanogenesis](http://projectreporter.nih.gov/project_info_description.cfm?icde=0&aid=8456852)* | Non-melanoma skin cancer | $331,957 | Yes |
|  | [Transcriptional Co-Regulatiors in Epidermis](http://projectreporter.nih.gov/project_info_description.cfm?icde=0&aid=8476898) | General cutaneous research | $319,730 | Yes |
|  | [Circadian Clock Regulation in Skin](http://projectreporter.nih.gov/project_info_description.cfm?icde=0&aid=8624582) | Non-melanoma skin cancer | $326,162 | Yes |
|  | [Phosphoinositide Signaling Regulates Melanogenesis](http://projectreporter.nih.gov/project_info_description.cfm?icde=0&aid=8683283)* | Non-melanoma skin cancer | $48,532 | Yes |
|  | [Aldehyde dehydrogenase: A novel regulator of melanin biogenesis](http://projectreporter.nih.gov/project_info_description.cfm?icde=0&aid=8424830) | General cutaneous research | $123,919 | Yes |
| University of California Los Angeles | [Characterization of the Lysogenic Pathway in P. acnes Bacteriophages](http://projectreporter.nih.gov/project_info_description.cfm?icde=0&aid=8526192) | Acne vulgaris | $20,553 | Yes |
|  | [Regulation of Langerhans Cell Migration by Invariant Gamma Delta T Cells](http://projectreporter.nih.gov/project_info_description.cfm?icde=0&aid=8551372) | Other (Lupus erythematosus) | $124,470 | Yes |
|  | [Immunobiology of Leprosy](http://projectreporter.nih.gov/project_info_description.cfm?icde=0&aid=8531865) | Leprosy | $1,409,483 | Yes |
|  | [CD1-restricted T Cell Responses in Skin](http://projectreporter.nih.gov/project_info_description.cfm?icde=0&aid=8535076) | Leprosy | $503,165 | Yes |
|  | [Innate Immunity in Acne Vulgaris](http://projectreporter.nih.gov/project_info_description.cfm?icde=0&aid=8424319) | Acne vulgaris | $244,717 | Yes |
|  | [Dermatology Scientist Training Program](http://projectreporter.nih.gov/project_info_description.cfm?icde=0&aid=8514525) | Training & department/institute program | $172,328 | No |
| University of California San Francisco | [Identification of Causal Variants in Psoriasis](http://projectreporter.nih.gov/project_info_description.cfm?icde=0&aid=8584976) | Psoriasis | $465,839 | Yes |
|  | Generation and maintenance of effector and regulatory T cells in the skin | General cutaneous research | $78,500 | Yes |
|  | [Genetic Analysis of the TNF Pathway in Psoriasis](http://projectreporter.nih.gov/project_info_description.cfm?icde=0&aid=8598626)* | Psoriasis | $1,000 | Yes |
|  | [Genetic Analysis of the TNF Pathway in Psoriasis](http://projectreporter.nih.gov/project_info_description.cfm?icde=0&aid=8507548)* | Psoriasis | $119,610 | Yes |
|  | [Mechanisms of immune regulation in the skin](http://projectreporter.nih.gov/project_info_description.cfm?icde=0&aid=8448638) | General cutaneous research | $125,685 | Yes |
|  | [Patient-Oriented Research in Skin Diseases](http://projectreporter.nih.gov/project_info_description.cfm?icde=0&aid=8528470) | Non-melanoma skin cancer | $182,524 | No |
|  | [Hedgehog signaling at the cell's antenna: Smoothened and the primary cilium](http://projectreporter.nih.gov/project_info_description.cfm?icde=0&aid=8500569) | General cutaneous research | $293,915 | Yes |
|  | [Cytokine Signaling in Neurons: Inflammation, Pain and Pruritis](http://projectreporter.nih.gov/project_info_description.cfm?icde=0&aid=8501382) | Pruritus | $330,244 | Yes |
|  | [UCSF Dermatology Training Grant](http://projectreporter.nih.gov/project_info_description.cfm?icde=0&aid=8464633) | Training & department/institute program | $202,247 | No |
| University of Chicago | [Coordinated cytoskeletal dynamics in skin somatic stem cells - Resubmission 01](http://projectreporter.nih.gov/project_info_description.cfm?icde=0&aid=8625508) | General cutaneous research | $348,394 | Yes |
|  | [Epidemic CA-MRSA: Molecular Epidemiology and Immunology](http://projectreporter.nih.gov/project_info_description.cfm?icde=0&aid=8457141) | Bacterial skin diseases | $320,112 | Yes |
| University of Cincinnati | [Coordinated Regulation of Hair Growth and Pigmentation by Dermal Papilla Cells](http://projectreporter.nih.gov/project_info_description.cfm?icde=0&aid=8518166) | General cutaneous research | $74,575 | Yes |
| University of Colorado | [MicroRNA-mediated Regulation in Mammalian Skin](http://projectreporter.nih.gov/project_info_description.cfm?icde=0&aid=8488415) | General cutaneous research | $306,508 | Yes |
| University of Colorado Denver | [Genetic studies of vitiligo](http://projectreporter.nih.gov/project_info_description.cfm?icde=0&aid=8578283) | Other (Vitiligo) | $446,579 | Yes |
|  | [Targeting p53-Dependent Repigmentation in Vitiligo](http://projectreporter.nih.gov/project_info_description.cfm?icde=0&aid=8510581) | Other (Vitiligo) | $100,572 | Yes |
|  | [Molecular Analysis, Modeling and Correction of Skin Diseases](http://projectreporter.nih.gov/project_info_description.cfm?icde=0&aid=8519055) | Training & department/institute program | $596,840 | No |
|  | [Vitiligo](http://projectreporter.nih.gov/project_info_description.cfm?icde=0&aid=8534707) | Other (Vitiligo) | $386,021 | Yes |
|  | [Testing the Therapeutic Potential of iPS Cells for Inherited Skin Diseases](http://projectreporter.nih.gov/project_info_description.cfm?icde=0&aid=8546231) | Other (Pemphigoid) | $327,661 | Yes |
|  | [The consequences of loricrin deficiency on epidermal barrier function](http://projectreporter.nih.gov/project_info_description.cfm?icde=0&aid=8488416) | Dermatitis including eczema  Other (Pemphigoid) | $313,443 | No |
|  | [The role of p63 in hair follicle stem cells and cancer](http://projectreporter.nih.gov/project_info_description.cfm?icde=0&aid=8489109) | Non-melanoma skin cancer | $318,881 | Yes |
| University of Iowa | [Regulation of the melanocyte lineage by the AP2 transcription factor family](http://projectreporter.nih.gov/project_info_description.cfm?icde=0&aid=8506691) | General cutaneous research | $319,972 | Yes |
|  | [Controlling the Mechanobiology of Cutaneous Wounds to Reduce Hypertrophic Scar](http://projectreporter.nih.gov/project_info_description.cfm?icde=0&aid=8583203) | Other (Hypertrophic disorders of skin) | $75,500 | Yes |
| University of Kentucky | [Non-contact Diffuse Optical Assessment of Pressure Ulcer and Therapy](http://projectreporter.nih.gov/project_info_description.cfm?icde=0&aid=8425042) | Decubitus ulcer | $156,387 | No |
| University of Maryland Baltimore | [Keratoderma- an Exploratory Study](http://projectreporter.nih.gov/project_info_description.cfm?icde=0&aid=8588676) | Other (Other epidermal thickening) | $163,094 | Yes |
| University of Michigan | [Biologic Activities of IL-36 Cytokines in Psoriasis](http://projectreporter.nih.gov/project_info_description.cfm?icde=0&aid=8563855) | Psoriasis | $101,142 | Yes |
|  | [The Role of Innate Immunity in Systemic and Cutaneous Lupus](http://projectreporter.nih.gov/project_info_description.cfm?icde=0&aid=8423448) | Other (Lupus erythematosus) | $128,232 | Yes |
|  | [Functional Genomics of Psoriasis](http://projectreporter.nih.gov/project_info_description.cfm?icde=0&aid=8584350) | Psoriasis | $470,734 | Yes |
|  | [Mechanisms of Altered Skin Re-Epithelialization in Aging](http://projectreporter.nih.gov/project_info_description.cfm?icde=0&aid=8502171) | General cutaneous research | $96,760 | Yes |
|  | [Role of the Psoriasis Associated IL23R Risk Variants on Th17 Biology and Function](http://projectreporter.nih.gov/project_info_description.cfm?icde=0&aid=8455694) | Psoriasis | $126,900 | Yes |
|  | [Characterizing the Cells of Origin for Basal Cell Carcinoma](http://projectreporter.nih.gov/project_info_description.cfm?icde=0&aid=8502244) | Non-melanoma skin cancer | $233,460 | Yes |
|  | [Linkage Analysis of Familial Psoriasis](http://projectreporter.nih.gov/project_info_description.cfm?icde=0&aid=8535074) | Psoriasis | $592,896 | Yes |
|  | [Molecular Regulation of Hair Follicle Morphogenesis](http://projectreporter.nih.gov/project_info_description.cfm?icde=0&aid=8460764) | General cutaneous research | $319,086 | Yes |
|  | [Role of Mast Cells in NLRP3-Mediated Skin Inflammation](http://projectreporter.nih.gov/project_info_description.cfm?icde=0&aid=8506980) | General cutaneous research | $319,086 | Yes |
|  | [TRP Ca2+ Channels in the Skin](http://projectreporter.nih.gov/project_info_description.cfm?icde=0&aid=8529459) | Non-melanoma skin cancer | $322,870 | Yes |
| University of Minnesota | [Skin-targeted Cell Therapy for Recessive Dystrophic Epidermolysis Bullosa](http://projectreporter.nih.gov/project_info_description.cfm?icde=0&aid=8502074) | Other (Pemphigoid) | $458,962 | Yes |
|  | [Role of Langerhans Cells in the Cutaneous Immune System](http://projectreporter.nih.gov/project_info_description.cfm?icde=0&aid=8443441) | General cutaneous research | $299,937 | Yes |
|  | [Regulated Activation of Latent-TGFb Determines Langerhans Cell Migration](http://projectreporter.nih.gov/project_info_description.cfm?icde=0&aid=8508067) | General cutaneous research | $322,763 | Yes |
|  | [Identification of a Keratinocyte Stem Cell Regulatory Gene in the KSC2 Locus](http://projectreporter.nih.gov/project_info_description.cfm?icde=0&aid=8508187) | General cutaneous research | $326,539 | Yes |
| University of Pennsylvania | [Psoriasis and the risk of diabetes](http://projectreporter.nih.gov/project_info_description.cfm?icde=0&aid=8486996) | Psoriasis | $162,474 | No |
|  | [HDAC functions in skin development, renewal and disease](http://projectreporter.nih.gov/project_info_description.cfm?icde=0&aid=8505758) | Non-melanoma skin cancer | $340,000 | Yes |
|  | [Hair follicle neogenesis in response to wounding](http://projectreporter.nih.gov/project_info_description.cfm?icde=0&aid=8505685) | General cutaneous research | $340,000 | Yes |
|  | [Migration of Skin Antibody Secreting Cells](http://projectreporter.nih.gov/project_info_description.cfm?icde=0&aid=8510380) | General cutaneous research | $53,942 | Yes |
|  | [Core Center](http://projectreporter.nih.gov/project_info_description.cfm?icde=0&aid=8499260) | Training & department/institute program | $608,000 |  |
|  | [Cloning and genetics of human pemphigus autoantibodies](http://projectreporter.nih.gov/project_info_description.cfm?icde=0&aid=8501378) | Other (Pemphigus) | $342,000 | Yes |
|  | [Characterization of neural crest stem cells in human hair follicles.](http://projectreporter.nih.gov/project_info_description.cfm?icde=0&aid=8449119) | General cutaneous research | $319,958 | Yes |
|  | [Regulation of T cell egress from inflamed skin](http://projectreporter.nih.gov/project_info_description.cfm?icde=0&aid=8477130) | General cutaneous research | $325,037 | Yes |
|  | [Filaggrin Mutations and the Prognosis of Atopic Dermatitis](http://projectreporter.nih.gov/project_info_description.cfm?icde=0&aid=8242639) | Dermatitis including eczema | $297,498 | No |
|  | [Role of autoantibody isotype in pemphigus pathogenesis](http://projectreporter.nih.gov/project_info_description.cfm?icde=0&aid=8449190) | Other (Pemphigus) | $334,703 | Yes |
|  | [WNT signals in skin and hair development and hair growth](http://projectreporter.nih.gov/project_info_description.cfm?icde=0&aid=8507841) | General cutaneous research | $342,000 | Yes |
|  | [Dermatology Research Training Grant](http://projectreporter.nih.gov/project_info_description.cfm?icde=0&aid=8468116) | Training & department/institute program | $280,794 | No |
| University of Pittsburgh at Pittsburgh | [Investigating the Neural Circuits of Itch](http://projectreporter.nih.gov/project_info_description.cfm?icde=0&aid=8417905) | Pruritus | $324,063 | Yes |
|  | [Using dual intersectional genetics to understand and modulate itch](http://projectreporter.nih.gov/project_info_description.cfm?icde=0&aid=8490842) | Pruritus | $194,438 | Yes |
|  | [T-helper cell cytokine profile in localized scleroderma](http://projectreporter.nih.gov/project_info_description.cfm?icde=0&aid=8598693)* | Other (Other localized connective tissue disorders) | $693 | Yes |
|  | [T-helper cell cytokine profile in localized scleroderma](http://projectreporter.nih.gov/project_info_description.cfm?icde=0&aid=8528476)* | Other (Other localized connective tissue disorders) | $125,679 | Yes |
|  | [The role of Atoh1 in the development and function of Merkel cells](http://projectreporter.nih.gov/project_info_description.cfm?icde=0&aid=8490166) | General cutaneous research | $308,836 | Yes |
| University of Rochester | [NF kappa B Activity in Skin Inflammation and Carcinogenesis](http://projectreporter.nih.gov/project_info_description.cfm?icde=0&aid=8514524) | General cutaneous research | $120,922 | Yes |
|  | [Training Grant in Dermatology](http://projectreporter.nih.gov/project_info_description.cfm?icde=0&aid=8467998) | Training & department/institute program | $212,813 | No |
| University of Southern California | [Development and Regeneration of Skin Appendages](http://projectreporter.nih.gov/project_info_description.cfm?icde=0&aid=8464525) | General cutaneous research | $439,908 | Yes |
|  | [Pattern Formation During Skin Organogenesis](http://projectreporter.nih.gov/project_info_description.cfm?icde=0&aid=8543624) | General cutaneous research | $350,639 | Yes |
|  | [Development of Extracellular Heat Shock Protein-90 as a Novel Topical Wound Heali](http://projectreporter.nih.gov/project_info_description.cfm?icde=0&aid=8461910) | General cutaneous research | $346,275 | Yes |
|  | [Tissue Engineering of New Hair Formation](http://projectreporter.nih.gov/project_info_description.cfm?icde=0&aid=8530017) | General cutaneous research | $332,424 | Yes |
|  | [Activator Inhibitor interactions in the cyclic regeneration of hair follicle stem](http://projectreporter.nih.gov/project_info_description.cfm?icde=0&aid=8497629) | General cutaneous research | $350,550 | Yes |
|  | [Isolation and characterization of new adult stem cells from sweat glands](http://projectreporter.nih.gov/project_info_description.cfm?icde=0&aid=8454545) | General cutaneous research | $76,950 | Yes |
| University of Tennessee Health Sci Ctr | [Role of exogenous melatonin in skin biology](http://projectreporter.nih.gov/project_info_description.cfm?icde=0&aid=8476985) | General cutaneous research | $316,350 | Yes |
| University of Toledo Health Sci Campus | [Regulation of Melanocyte Differentiation by SWI/SNF Chromatin Remodeling Enzymes](http://projectreporter.nih.gov/project_info_description.cfm?icde=0&aid=8471062) | General cutaneous research | $307,390 | Yes |
| University of Virginia | [Regulation of TSLP receptor expression and function in eczema in mice and man](http://projectreporter.nih.gov/project_info_description.cfm?icde=0&aid=8460063) | Dermatitis including eczema | $515,908 | Yes |
| University of Washington | [The replication checkpoint and genomic fidelity in skin](http://projectreporter.nih.gov/project_info_description.cfm?icde=0&aid=8438257) | Non-melanoma skin cancer | $334,455 | Yes |
|  | [Keratin Gene Targeting for the Treatment of Epidermolysis Bullosa](http://projectreporter.nih.gov/project_info_description.cfm?icde=0&aid=8401502) | Other (Pemphigoid) | $302,236 | Yes |
|  | [Training for Investigative Dermatology](http://projectreporter.nih.gov/project_info_description.cfm?icde=0&aid=8494572) | Training & department/institute program | $148,770 | No |
| University of Wisconsin-Madison | [RNA-binding protein CRD-BP in melanocyte biology](http://projectreporter.nih.gov/project_info_description.cfm?icde=0&aid=8437734) | General cutaneous research | $316,379 | Yes |
|  | [18th Annual Meeting of the PanAmerican Society of Pigment Cell Research](http://projectreporter.nih.gov/project_info_description.cfm?icde=0&aid=8597622) | Conference | $24,000 | No |
|  | [Role of Polo-Like Kinase-1 in Melanocytic Transformation](http://projectreporter.nih.gov/project_info_description.cfm?icde=0&aid=8492041) | Melanoma | $303,130 | Yes |
|  | [Caspase-14 and the Treatment of Psoriasis](http://projectreporter.nih.gov/project_info_description.cfm?icde=0&aid=8509601) | Psoriasis | $304,722 | Yes |
|  | [Autophagy in epidermal melanocyte: a protective or a destructive role?](http://projectreporter.nih.gov/project_info_description.cfm?icde=0&aid=8496721) | General cutaneous research | $164,913 | Yes |
|  | [The Cutaneous Biology of MAGE Transcription Factors](http://projectreporter.nih.gov/project_info_description.cfm?icde=0&aid=8497632) | Melanoma | $160,847 | No |
|  | [Identification of Dysregulated Stress Response Mechanisms in Chronic Urticaria](http://projectreporter.nih.gov/project_info_description.cfm?icde=0&aid=8543629) | Urticaria | $193,016 | No |
|  | [Investigative Dermatology Training Program at University of Wisconsin-Madison](http://projectreporter.nih.gov/project_info_description.cfm?icde=0&aid=8461168) | Training & department/institute program | $220,580 | No |
| UT MD Anderson Cancer Ctr | [Keratinocyte Activation by Slug-induced Calprotectin](http://projectreporter.nih.gov/project_info_description.cfm?icde=0&aid=8432042) | General cutaneous research | $202,635 | Yes |
| UT Southwestern Medical Center | [Molecular Markers in Discoid Lupus Erythematous](http://projectreporter.nih.gov/project_info_description.cfm?icde=0&aid=8500214) | Other (Lupus erythematosus) | $123,012 | Yes |
|  | [Striatal Dopamine Release in Response to Ultraviolet Light in Compulsive Tanners](http://projectreporter.nih.gov/project_info_description.cfm?icde=0&aid=8519310) | Other (Skin changes due to chronic exposure to nonionizing radiation) | $169,931 | No |
| Vanderbilt University Med Ctr | [Linking Lipoxygenases with Essential Fatty Acids and Epidermal Barrier Formation](http://projectreporter.nih.gov/project_info_description.cfm?icde=0&aid=8486396) | Other (Other epidermal thickening) | $333,450 | Yes |
|  | [Skin Regeneration with Stem Cells and Scaffolds](http://projectreporter.nih.gov/project_info_description.cfm?icde=0&aid=8508674) | General cutaneous research | $601,177 | Yes |
|  | [The role of microRNA miR-31 in skin biology](http://projectreporter.nih.gov/project_info_description.cfm?icde=0&aid=8481191) | General cutaneous research | $333,450 | Yes |
| Wake Forest University Health Sciences | [Regulation of Itch Scratching by Spinal GRP Receptors in Primates](http://projectreporter.nih.gov/project_info_description.cfm?icde=0&aid=8492849) | Pruritus | $195,075 | Yes |
|  | [Behavioral Pharmacology of Itch](http://projectreporter.nih.gov/project_info_description.cfm?icde=0&aid=8521083) | Pruritus | $314,469 | Yes |
| Washington University | [Smart Laser Treatment of Port-Wine Stain in Children](http://projectreporter.nih.gov/project_info_description.cfm?icde=0&aid=8515337) | Miscellaneous | $86,865 | No |
|  | [Molecular Mechanisms of the Itch Sensation in the Spinal Cord](http://projectreporter.nih.gov/project_info_description.cfm?icde=0&aid=8453255) | Pruritus | $308,785 | Yes |
| Yale University | [Skin Development Analysis in Mutant Mouse Lines](http://projectreporter.nih.gov/project_info_description.cfm?icde=0&aid=8460927) | General cutaneous research | $355,716 | Yes |
|  | [Extrinsic Regulation of Epidermal Homeostatasis](http://projectreporter.nih.gov/project_info_description.cfm?icde=0&aid=8460545) | General cutaneous research | $355,716 | Yes |
|  | [Mechanisms of Genetic Reversion in Ichthyosis With Confetti](http://projectreporter.nih.gov/project_info_description.cfm?icde=0&aid=8522261) | Other (Other epidermal thickening) | $355,894 | Yes |
|  | [Live Imaging of Skin Regeneration](http://projectreporter.nih.gov/project_info_description.cfm?icde=0&aid=8541695) | General cutaneous research | $355,894 | Yes |
|  | [Dermatology Training Grant](http://projectreporter.nih.gov/project_info_description.cfm?icde=0&aid=8501372) | Training & department/institute program | $226,521 | No |

* Distinct grants despite having same title

**Table S2. 15 Skin conditions studied by GBD 2010 and grant title or abstract terms supporting inclusion**

| **Skin Condition (ICD-10 codes)^a^** | **Grant Title or Abstract Terms** |
| --- | --- |
| Dermatitis including eczema (L20-L27)^b^ | “eczema” “dermatitis” “atopic dermatitis” “contact dermatitis” “irritant contact dermatitis”  “allergic contact dermatitis” “exfoliative dermatitis” “diaper dermatitis” “seborrheic dermatitis” |
| Acne vulgaris (L70) | “acne vulgaris” “acne” |
| Bacterial skin diseases (L00,L01, L02, L04, L08, L88,L97, L98.0-L98.4) | “staphylococcal scalded skin syndrome” “impetigo” “cutaneous abscess/furuncle/ carbuncle” “lymphadenitis” “pyoderma” “erythrasma” “bacteria skin” |
| Viral skin diseases (B00, B07-B09) | “herpes” “viral warts” “molluscum contagiosum” “exanthema subitum” “viral” and “skin” |
| Urticaria (L50) | “urticaria” |
| Fungal skin diseases (B35, B36.0, B36.1, B36.2, B36.3, B36.8, B36.9) | “fungal” and “skin” “candidiasis” “tinea” |
| Pruritus (L29) | “pruritus” “itch” |
| Scabies (B66) | “scabies” |
| Alopecia areata (L63.0, L63.1, L63.8, L63.9) | “alopecia areata” |
| Cellulitis (L03.0, L03.1, L03.2-L03.9) | “cellulitis” |
| Decubitus ulcer (L89) | “decubitus ulcer” “pressure wound” |
| Melanoma (C43) | “melanoma” |
| Psoriasis (L40, L41) | “psoriasis” |
| Non-melanoma skin cancer (C44, D04) | “non-melanoma skin cancer” “basal cell carcinoma” “squamous cell carcinoma” |
| Leprosy (A30) | “leprosy” |

^a^ See reference 11

*excluded L28 (lichen simplex chronicus and prurigo)

**Table S3. “Other skin and subcutaneous diseases” studied by GBD 2010 and grant title or abstract terms supporting inclusion**

| **GBD “other skin and subcutaneous diseases” category (ICD-10 code)^a^** | **Abstract/project terms supporting inclusion** |
| --- | --- |
| Pediculosis and phthiriasis (B85) | “pediculosis” “phthiriasis” |
| Myiasis (B87) | “myiasis” |
| Other infestations (B88) | “ acariasis” “tungiasis” or “sandflea” “scarabiasis” or “arthropod” “hirudiniasis” or “leech” “ichthyoparasitism” “vandellia cirrhosa” “linguatulosis” “porocephaliasis” “skin infestation” “mites” “skin parasites” |
| Pilonidal cyst with and without abscess (L05.0 and L05.9) | “pilonidal cyst” |
| Pemphigus (L10) | “pemphigus” |
| Other acantholytic disorders (L11) | “keratosis follicularis” “acantholytic dermatosis” “acantholytic” |
| Pemphigoid (L12) | “pemphigoid” “bullous” or “bulla” “epidermolysis bullosa” |
| Other bullous disorders (L13) | “dermatitis herpetiformis” “subcorneal pustular dermatitis” “bullous” “bulla” |
| Lichen simplex chronicus and prurigo (L28) | “lichen simplex chronicus” “prurigo” |
| Other dermatitis (L30) | “nummular dermatitis” “dyshidrosis” “cutaneous autosensitization” “infective dermatitis” “erythema intertrigo” “pityriasis alba” |
| Pityriasis rosea (L42) | “pityriasis rosea” |
| Lichen planus (l42) | “lichen planus” |
| Other papulosquamous disorders (L44) | “pityriasis rubra pilaris” “lichen nitidus” “lichen striatus” “lichen ruber moniliformis” “infantile papular acrodermatitis” or “Giannotti-Crosti” “papulosquamous” |
| Erythema multiforme (L51) | “erythema multiforme” “Stevens Johnson” |
| Erythema nodosum (L52) | “erythema nodosum” |
| Other erythematous conditions (L53) | “toxic erythema” “erythema annulare centrifugum” “erythema marginatum” “chronic figurate erythema” |
| Sunburn (L55) | “sunburn” |
| Other acute skin changes due to ultraviolet radiation (L56) | “drug phototoxic” “drug photoallergic” “photocontact dermatitis” “solar urticaria” “polymorphous light eruption” “acute” and “UV” or “ultraviolet” |
| Skin changes due to chronic exposure to nonionizing radiation (L57) | “actinic keratosis” “actinic reticuloid” “cutis rhomboidalis nuchae” “poikiloderma of Civatte” “cutis laxa senilis” “actinic granuloma” “nonionizing radiation” “wrinkles” “chronic” and “UV” or “ultraviolet” “tanning” |
| Radiodermatitis (L58) | “radiodermatitis” |
| Other disorders of skin and subcutaneous tissue related to radiation (L59) | “erythema ab igne” “dermatitis ab igne” |
| Nail disorder (L60) | “nail” “ingrowing” “onycholysis” “onychogryphosis” “Beau lines” “yellow nail syndrome” |
| Androgenic alopecia (L64) | “androgenic alopecia” |
| Other nonscarring hair loss (L65) | “telogen effluvium” “anagen effluvium” “alopecia mucinosa” “nonscarring” “hair loss” “alopecia” |
| Cicatricial alopecia (scarring hair loss) (L66) | “pseudopelade” “lichen planopilaris” “folliculitis decalvans” “perifolliculitis capitis abscedens” “folliculitis ulerythematosa reticulate” “cicatricial” “scarring” “hair loss” “alopecia” |
| Hair color and hair shaft abnormalities (L67) | “trichorrhexis nodosa” “hair color” “hair shaft” |
| Hypertrichosis  (L68) | “hirsutism” “hypertrichosis” “polytrichia” |
| Rosacea (L71) | “rosacea” “perioral dermatitis” “rhinophyma” |
| Follicular cysts of skin and subcutaneous tissue (L72) | “cyst” “follicular” “epidermal” “trichilemmal” “steatocystoma” |
| Other follicular disorders (L73) | “acne keloid” “pseudofolliculitis barbae” “hidradenitis suppurativa” |
| Eccrine sweat disorders (L74) | “eccrine sweat” “miliaria” “anhydrosis” |
| apocrine sweat disorder (L75) | “apocrine sweat” “bromhidrosis” “chromhidrosis”  “apocrine miliaria” |
| Vitiligo (L80) | “vitiligo” |
| Other disorders of pigmentation (L81) | “hyperpigmentation” “chloasma” “freckles” “café au lait spots” “leukoderma” “hypopigmentation” [excluding “vitiligo] “pigmented purpuric dermatosis” “iron pigmentation” “tattoo pigmentation” |
| Seborrhoeic keratosis (L82) | “seborrhoeic keratosis” |
| Acanthosis nigricans (L83) | “acanthosis nigricans” |
| Corns and callosities (L84) | “corn” “callous” |
| Other epidermal thickening (L85) | “(acquired) icthyosis” “keratoderma” “(acquired)keratosis” “xerosis cutis” “epidermal thickening” |
| Transepidermal elimination disorders (L87) | “keratosis follicularis et parafollicularis in cutem penetrans” “reactive perforating collagenosis” “elastosis perforans serpiginosa” “transepidermal” |
| Atrophic disorders of skin (L90) | “lichen sclerosis” “anetoderma” “atrophoderma” “acrodermatitis chronica atrophicans” “adherent scar” “cicatrix” “striae atrophicae” |
| Hypertrophic disorders of skin (L91) | “hypertrophic scar” “keloid” |
| Granulomatous disorders of skin and subcutaneous tissue (L92) | “granuloma annulare” “necrobiosis lipoidica” “granuloma faciale” “eosinophilic granuloma of skin” “foreign body granuloma” and “skin” |
| Lupus erythematosus (L93) | “discoid” “cutaneous” and “lupus” “local” and “lupus” |
| Other localized connective tissue disorders (L94) | “localized scleroderma” “morphea” “linear scleroderma” “calcinosis cutis” “sclerodactyly” “Gottron papules” “poikiloderma vasculare atrophicans” “ainhum” |
| L95: Vasculitis limited to skin, not elsewhere classified (L95) | “livedoid vascultitis” “erythema elevatum diutinum” “vasculitits” and “skin” |

^a^ See reference 11

**Table S4. Additional categories and grant title or abstract terms supporting inclusion**

| **Category** | **Abstract/project terms supporting inclusion** |
| --- | --- |
| Training & department/institution program | “dermatology” and “training” (excludes conference-specific training; excludes training of one individual outside of a designated program) |
| Conference | “skin/dermatology” and “conference/symposium” “retreat” dates and place of event given |
| Other miscellaneous skin diseases (not in other GBD category) | “pachyonychia congenita” “port wine stain” “hemangioma” “melanocytic nevi” vesicant induced skin injury including but not limited to: “lewsite-mediated cutaneous blistering-inflammation ” “nitrogen mustard-related nitrosureas” induced skin injury |
